# Supplementary material for: The emotional mechanism underlying the adverse effect of social exclusion on working memory performance: A tDCS study
Source: PLoS One. 2023 Apr 10;18(4):e0284262. doi: 10.1371/journal.pone.0284262 (PMC10085037; doi:10.1371/journal.pone.0284262)
Supplement: Attachment — Submitted filename: Response to Reviewers.docx [file pone.0284262.s002.docx]

Dear editor and reviewer,

We appreciate the valuable comments and suggestions given by the reviewer, which have been conscientiously considered in our amendment of the manuscript and significantly improve the manuscript. All the changes made to the manuscript are marked with Track Changes in the revised manuscript. Our detailed responses to the comments of the reviewers are given below.

Response to reviewer #1

Point 1. How did the authors determine the size sample? Did they use Gpower, did they refer to previous literature? I think they should add a short statement clarifying this aspect.

Response: Thanks for reviewer’s suggestions. The way we determined the size sample was according to the calculation of G*Power 3.1 (Faul et al., 2009). Under the premise of power (1-β) =0.95, α = 0.05 and effect size f = 0.4 (Weintraub-Brevda & Chua, 2019), a minimum of 84 participants were needed, with 21 in each group. As a reference, Riva et al. (2015) recruited 80 participants with 20 in each group and He et al. (2018) recruited 44 participants with 22 in each group. Based on this, 128 participants were recruited in our research with 32 in each group. A short statement clarifying how we determined the size sample is added in the Participants section (page 3) in the 'Revised Manuscript with Track Changes' file.

Reference:

[1] Faul, F., Erdfelder, E., Buchner, A., & Lang, A. G. Statistical power analyses using G* Power 3.1: Tests for correlation and regression analyses. Behavior research methods. 2009;41(4):1149-1160.

[2] Weintraub-Brevda, R. R., & Chua, E. F. Transcranial direct current stimulation over the right and left VLPFC leads to differential effects on working and episodic memory. Brain and Cognition. 2019;132:98-107.

Point 2. There is an aspect that a bit crucial to me: the difference in mood rating between excluded and included participants, after receiving anodal stimulation was marginally significant (p = 0.06). I consider that the authors should discuss this issue, since I don’t think that they can strongly claim that the anodal tDCS reduced the negative emotion induced by social exclusion compared to social inclusion. This result, consequently affects the conclusion that the performance in WM is mainly modulate by low mood rating. In fact, the main conclusion that the authors can state is that anodal tDCS reverse the effect of social exclusion on the WM, but they cannot conclude that such modulation is principally due to the mood rating.

Response: Thanks for reviewer’s suggestions. We agree with your opinion that we can’t strongly claim that the anodal tDCS reduced the negative emotion induced by social exclusion compared to social inclusion based on the marginally significant result (p = 0.06). It might indicate that the difference in mood rating score between excluded and included participants had a tendency to decrease under anodal tDCS. We have modified the description in the Result section (page 5) in the 'Revised Manuscript with Track Changes' file.

In order to probe the relationship between social exclusion, negative emotion and WM performance, we applied a mediation model to analyze the data from sham stimulation group through the PROCESS SPSS computational tool. The results indicated that the impairment of social exclusion on WM performance was fully mediated by negative emotion. Besides, we applied a 2 × 2 between-subject ANOVA to test the alleviating effect of anodal tDCS over the rVLPFC on the adverse effect of social exclusion on WM performance. The ANOVA results showed that mood rating score and 2-back task accuracy of excluded participants receiving anodal tDCS over the rVLPFC were both significantly higher than excluded participants receiving sham tDCS. Besides, we also analyzed the mediation model with the data from the anodal stimulation condition and found no more mediating effect. These results contributed to our second conclusion that anodal tDCS over the rVLPFC would alleviate the adverse effect of social exclusion on WM performance by reducing the negative emotion.

Point 3. A curiosity: why the authors only considered to test the excitatory effect of the stimulation, and they didn’t take into account the inhibitory aspect, delivering cathodal tDCS over the rVLPFC?

Response: Thanks for reviewer’s suggestions. One of the aims of this study was to investigate the potential solution of neural intervention to reduce the impairment of social exclusion on working memory performance. Based on the findings from previous studies that (1) social exclusion would induce negative emotion (e.g., anxiety, sadness, depression), and (2) negative emotion would impair WM performance, we first hypothesized that negative emotions induced by social exclusion mediate the relationship between social exclusion and WM performance. Besides, according to the findings from previous studies that anodal stimulation over the rVLPFC would reduce the negative emotion, we further hypothesized that anodal stimulation over the rVLPFC would alleviate the adverse effect of social exclusion on WM performance. Along this line, we only considered to test the excitatory effect of the stimulation because it was in line with our study purpose i.e. investigate the potential solution of neural intervention to alleviate the impairment of social exclusion on WM performance.

According to reviewer’s suggestion and for further understanding the role of rVLPFC in the impairment of social exclusion on WM performance, we will take cathodal tDCS into account to test the inhibitory aspect in our future research. We have added this future research direction in the Discussion section (page 9) in the 'Revised Manuscript with Track Changes' file.

Sincerely,

Qingguo Ma on behalf of the co-authors.
